# Exploring Biomarkers of Oxidative Stress in Health and Disease: Editorial Overview

**DOI:** 10.3390/antiox14121400

**Published:** 2025-11-25

**Authors:** Denisa Margina, Daniela Gradinaru

**Affiliations:** Faculty of Pharmacy, Carol Davila University of Medicine and Pharmacy, 6 Traian Vuia Street, 020956 Bucharest, Romania

## 1. Introduction

Over the past four decades, the concept of oxidative stress has remained a cornerstone of biomedical research. Defined as an imbalance between the production of reactive oxygen species (ROS) and the capacity of antioxidant defenses to neutralize them, oxidative stress is now recognized as both a physiological signaling mechanism and a pathological driver of disease. The disruption of redox homeostasis contributes to cumulative damage to DNA, proteins, and lipids, ultimately impairing cellular and tissue function. This redox imbalance has been involved in the development of numerous chronic conditions, including cardiovascular and metabolic diseases, cancer, neurodegenerative disorders, and autoimmune pathologies, as well as in the natural processes of aging.

Recent advances in the field have moved beyond the mere measurement of oxidative damage toward the identification of reliable biomarkers capable of reflecting dynamic redox states in health and disease. These biomarkers have become essential tools not only for unraveling mechanisms of pathogenesis but also for evaluating therapeutic efficacy and predicting disease outcomes. For instance, pharmacological modulation of redox signaling pathways—such as activation of the Nrf2-Keap1 axis—has emerged as a promising strategy in targeted therapies for inflammatory and neurodegenerative conditions. At the same time, the development of novel analytical technologies has refined the detection of oxidative stress-related molecules in clinical and experimental settings.

In this Special Issue, we bring together a collection of studies that collectively advance our understanding of oxidative stress biomarkers across diverse physiological, pathological, and toxicological contexts. The selected works span from clinical assessments and experimental mechanistic explorations to molecular genetics and therapeutic strategies, reflecting the broad translational potential of redox research.

## 2. Highlights of Contributions

### 2.1. Clinical and Translational Biomarker Studies

Several contributions in this issue focus on the clinical assessment of oxidative stress markers and their diagnostic or prognostic implications. A prospective investigation into inflammatory bowel disease (IBD) evaluated the diagnostic value of oxidative stress-related biomarkers—including ceruloplasmin, gamma-glutamyl transferase, and albumin—in distinguishing active disease from remission. The study demonstrated their potential as dual tools for assessing remission and predicting treatment outcomes, reinforcing the role of redox dysregulation in IBD pathophysiology [contribution 1].

Another clinical study characterized oxidative stress profiles in patients with long COVID [contribution 2] during the Omicron phase in Japan. Elevated diacron-reactive oxygen metabolites and oxidative stress indices were particularly associated with female sex, age, and metabolic status, and correlated with inflammatory markers such as C-reactive protein and fibrinogen. Notably, oxidative stress levels were higher in patients experiencing neurological symptoms such as brain fog, suggesting a link between redox imbalance and neurocognitive dysfunction in post-viral syndromes.

In the context of chronic obstructive pulmonary disease (COPD) [contribution 3], gene expression analyses revealed that exacerbation events are accompanied by altered mRNA expression of key enzymes in glutathione metabolism and inflammatory pathways. These results underscore the systemic nature of oxidative stress and inflammation in respiratory disorders.

### 2.2. Mechanistic and Experimental Insights

Complementing the clinical findings, several experimental studies explored molecular pathways underlying oxidative stress responses. The study on Sheng Mai San [contribution 4], a classical Traditional Chinese Medicine formulation, demonstrated that it mitigates heat stress-induced myocardial injury through coordinated regulation of the Keap1-Nrf2-HO-1 and Stub1-HSF1 signaling pathways. This work illustrates how natural compounds can exert multi-targeted antioxidant and cytoprotective effects.

In the same framework, a study on selenium-binding protein 1 (SELENBP1) revealed that its deficiency in dendritic cells confers protection against sepsis by modulating the Treg/Th17 balance and dampening inflammatory injury [contribution 5]. These mechanistic insights illuminate how redox-sensitive signaling molecules influence immune responses and disease outcomes.

In another experimental context, a newly developed immunoassay [contribution 6] was introduced to quantify the neutralization capacity of plasma against oxidation-specific epitopes. This assay provides a promising approach to assess the functional ability of biological fluids to counteract lipid peroxidation products, potentially offering a novel biomarker for oxidative resilience.

### 2.3. Molecular and Genetic Perspectives

Several papers extended the exploration of oxidative stress into the genomic and molecular domains. Data from the European MARK-AGE [contribution 7] study applied causal inference models to uncover relationships between LDL oxidation, nitric oxide metabolism, telomere length, and DNA integrity—highlighting oxidative stress as a central determinant of genomic stability and vascular aging.

Additionally, research on Mexican children [contribution 8] demonstrated that single-nucleotide polymorphisms in antioxidant genes such as SOD2 and GPX1 modulate the relationship between oxidative stress markers and obesity-related phenotypes. These findings emphasize the importance of genetic background in shaping redox homeostasis from early life stages.

At the interface of oncology and immunotherapy, a review on oxidized low-density lipoprotein (oxLDL) proposed its role as a mediator of immune checkpoint inhibitor resistance in microsatellite-stable colorectal cancer. By influencing tumor metabolism and immune cell dynamics, oxLDL emerges as a potential therapeutic target for overcoming immunotherapy resistance [contribution 9].

### 2.4. Emerging Perspectives and Challenges

Collectively, the studies in this Special Issue reinforce the growing recognition that oxidative stress is not merely a byproduct of disease but a central mechanism influencing susceptibility, progression, and response to therapy. The integration of redox biomarkers into clinical practice remains challenging due to biological variability, methodological inconsistencies, and the complex interplay between oxidative and antioxidant systems [contribution 10].

Future research must therefore focus on the standardization of biomarker assays, longitudinal validation in diverse populations, and systems-level modeling of redox networks. Furthermore, cross-disciplinary approaches that link redox biology with omics technologies and artificial intelligence could enable more precise disease stratification and personalized therapeutic strategies.

## 3. Conclusions

The contributions assembled in this Special Issue collectively advance the frontier of oxidative stress research by providing novel methodological, mechanistic, and translational insights. They highlight oxidative stress biomarkers as promising tools for diagnosis, prognosis, and therapeutic monitoring across a spectrum of diseases. As the field progresses, the convergence of clinical, molecular, and computational approaches will be pivotal for translating redox biology into tangible clinical impact.

